# Lack of UBE3A-Mediated Regulation of Synaptic SK2 Channels Contributes to Learning and Memory Impairment in the Female Mouse Model of Angelman Syndrome

**DOI:** 10.1155/2022/3923384

**Published:** 2022-10-04

**Authors:** Jiandong Sun, Yan Liu, Xiaoning Hao, Michel Baudry, Xiaoning Bi

**Affiliations:** ^1^College of Osteopathic Medicine of the Pacific, Western University of Health Sciences, Pomona, California 91766, USA; ^2^College of Dental Medicine, Western University of Health Sciences, Pomona, California 91766, USA

## Abstract

Angelman syndrome (AS) is a rare neurodevelopmental disorder characterized by severe developmental delay, motor impairment, language and cognition deficits, and often with increased seizure activity. AS is caused by deficiency of UBE3A, which is both an E3 ligase and a cofactor for transcriptional regulation. We previously showed that the small conductance potassium channel protein SK2 is a UBE3A substrate, and that increased synaptic SK2 levels contribute to impairments in synaptic plasticity and fear-conditioning memory, as inhibition of SK2 channels significantly improved both synaptic plasticity and fear memory in male AS mice. In the present study, we investigated UBE3a-mediated regulation of synaptic plasticity and fear-conditioning in female AS mice. Results from both western blot and immunofluorescence analyses showed that synaptic SK2 levels were significantly increased in hippocampus of female AS mice, as compared to wild-type (WT) littermates. Like in male AS mice, long-term potentiation (LTP) was significantly reduced while long-term depression (LTD) was enhanced at hippocampal CA3-CA1 synapses of female AS mice, as compared to female WT mice. Both alterations were significantly reduced by treatment with the SK2 inhibitor, apamin. The shunting effect of SK2 channels on NMDA receptor was significantly larger in female AS mice as compared to female WT mice. Female AS mice also showed impairment in both contextual and tone memory recall, and this impairment was significantly reduced by apamin treatment. Our results indicate that like male AS mice, female AS mice showed significant impairment in both synaptic plasticity and fear-conditioning memory due to increased levels of synaptic SK2 channels. Any therapeutic strategy to reduce SK2-mediated inhibition of NMDAR should be beneficial to both male and female patients.

## 1. Introduction

Angelman syndrome (AS) is a rare neurodevelopmental disorder with an incidence of approximately 1 in 10,000 to 20,000 live births [1, 2]. AS is characterized by severe developmental delay, language and cognition deficits, motor dysfunction [3, 4], unusually happy demeanor, and in many AS patients, seizure activity and autism-like behavior [3, 5, 6]. AS is caused by the deficient expression of the maternally inherited *UBE3A* gene in neurons [7–13], because neuronal expression of the paternal allele is silenced by a long noncoding antisense RNA transcript (*UBE3A-ATS*) [14–19]. Common maternal UBE3A deficiency has been attributed to four genetic etiologies [10, 20]: deletions of the maternal 15q11–q13 region (class I, approximately 70% of cases), paternal uniparental disomy of chromosome 15 (class II, 5%), imprinting defects (class III, 5%), and mutations in *UBE3A* (class IV, 10%). The *UBE3A* gene encodes an E ligase, UBE3A, also known as E6-associated protein (E6AP), the founding member of the HECT (homologous to E6AP carboxy terminus) domain-containing E3 ligase family [21]. Although there is no clear sex difference in the incidence of AS, emerging evidence indicates that the clinical presentation and pathogenesis of the disease may differ between male and female patients. For instance, a study of 24 AS patients (11 males and 13 females) reported that a larger proportion of male patients could walk independently, as compared to female patients [22]. A more recent study with 110 adolescents and adults with AS reported that obesity disproportionately affected female AS patients [23]. However, due to the rare prevalence of the disease, sex differences in cognitive function have not been widely studied.

A few lines of transgenic mice with maternal UBE3a deficiency (AS mice) have been developed and tremendously contributed to our understanding of AS pathogenesis, as these mice exhibit several features of the human disease, including reduced brain size, abnormal electroencephalogram, learning and memory deficits, motor dysfunction [24–28], as well as impairment in long-term potentiation (LTP) of synaptic transmission [25, 29–33]. The influence of age and background strains on phenotypic syndromes and their severity across these mouse models has been well characterized [26, 34, 35]. While a few studies have also addressed sex differences [26, 34], their results are not conclusive, and clear sex differences were only observed in body weight [26], motor function, and nest building [34]. A more recent study specifically designed to compare male and female AS mice using several behavioral tests identified sex differences in some behavioral features, which were mainly due to differences in sensory perception [36]. We previously reported that the small conductance potassium channel protein SK2 is a UBE3a substrate, and that its synaptic levels are significantly increased in the hippocampus of male AS mice, an effect which contributes to LTP impairment [37, 38], and reduced memory recall in the fear-conditioning paradigm in these mice [37]. The critical roles of SK channels in LTP and in learning and memory in wild-type (WT) mice have been well documented in the literature [39–41]. We also showed that enhanced synaptic SK2 levels in AS mice imposed a stronger inhibition of NMDARs, resulting in impaired LTP and fear memory [37], which is in agreement with the literature [39, 40]. The degree of activation of NMDARs and the efficacy of their downstream signaling pathways have been shown to be different in adult male and female mice [42, 43]. Thus, the present studies were directed at evaluating synaptic SK2 levels, NMDAR regulation by SK2, and contextual and auditory fear memory in female AS mice.

## 2. Results

### 2.1. Levels of SK2 Channels Are Increased in the Hippocampus of Female UBE3A-Deficient Mice

**Figure 1:**
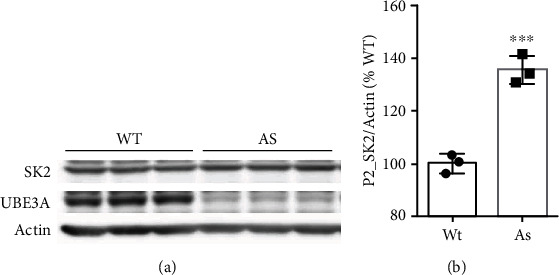
SK2 expression in hippocampus of female AS and WT mice. (a) Western blot analysis of SK2 levels in crude synaptosomal fractions (P2) fractions from hippocampus of female AS and WT mice. (b) Quantitative analysis of blots. Results are expressed as % of values in WT mice and shown as means ± SEM. *N* = 3 *mice*, ^∗∗∗^*P* < 0.001 (unpaired, two-tailed Student's t-test).

**Figure 2:**
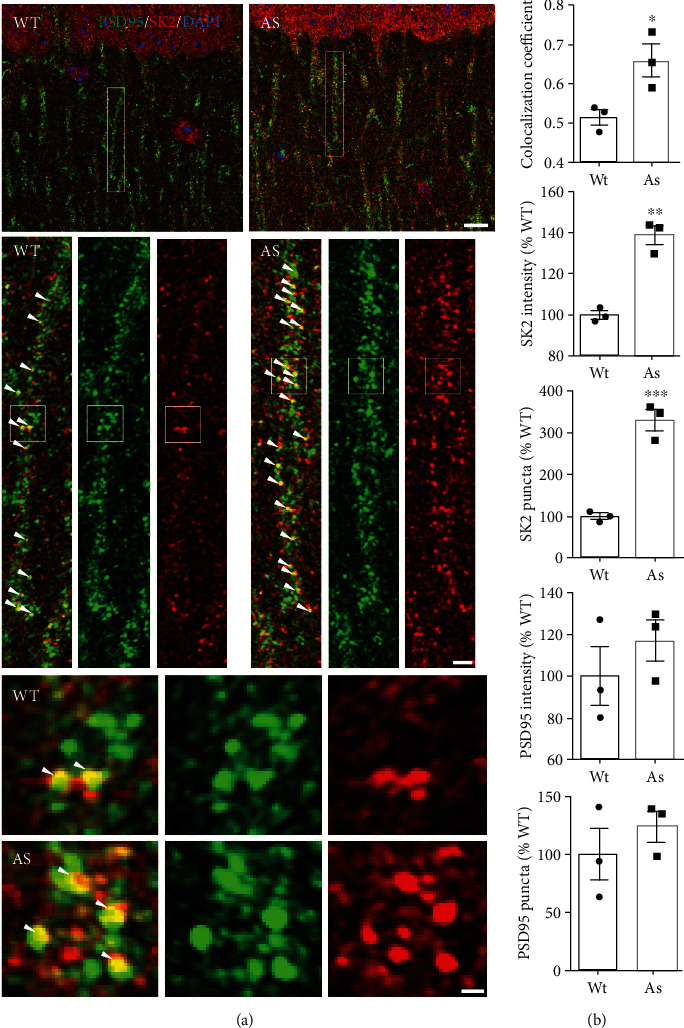
Localization of synaptic SK2 in hippocampal CA1 region of female AS and WT mice. (a) Representative images of CA1 pyramidal neurons stained with anti-SK2 (red) and -PSD95 (green) antibodies. Middle panels are enlarged images of boxed areas in top panels and bottom panels are enlarged images of boxed areas in middle panels. Arrowheads indicate clearly colocalized puncta. Top panels, scale bar = 10 *μ*m. Middle panels, scale bar = 2 *μ*m. Bottom panels, scale bar = 0.5 *μ*m. (b) Quantitative analysis of the intensity and number of SK2- and PSD95-immunoreactive puncta and SK2 and PSD95 colocalization in hippocampal CA1 region. *N* = 3 mice per group (unpaired t-test, ^∗^*P* < 0.05, ^∗∗^*P* < 0.01, ^∗∗∗^*P* < 0.001).

We first determined synaptic SK2 expression in the hippocampus of female mice. Western blot analysis of hippocampal proteins from female AS mice showed that SK2 levels were significantly increased in crude synaptosomal fractions, as compared to those from female WT mice (P2; Figure 1), a finding similar to what we previously reported for male AS mice. UBE3a deficiency was confirmed by western blot analysis (Figure 1). Immunofluorescent staining of brain sections showed prominent SK2 staining in hippocampal CA1 region, especially in cell bodies and dendrites of pyramidal neurons (Figure 2(a)). High-magnification examination revealed that both the intensity and numbers of SK2-immunoreactive puncta distributed along apical dendrites of CA1 pyramidal neurons were significantly increased in female AS mice as compared to female WT mice (Figures 2(a) and 2(b)). SK2-immunopositive puncta were partially colocalized with PSD95-immunopositive puncta in both WT and AS mice. Quantitative analysis showed that the percentage of puncta dually stained with SK2 and PSD95 antibodies was significantly increased in female AS mice, as compared to female WT mice (Figure 2(b)). There was no significant difference in the overall number of PSD95-immunopositive puncta between female AS and WT mice (Figure 2(b)).

### 2.2. Increased SK2 Levels Contribute to Synaptic Plasticity Impairment in Female AS Mice

**Figure 3:**
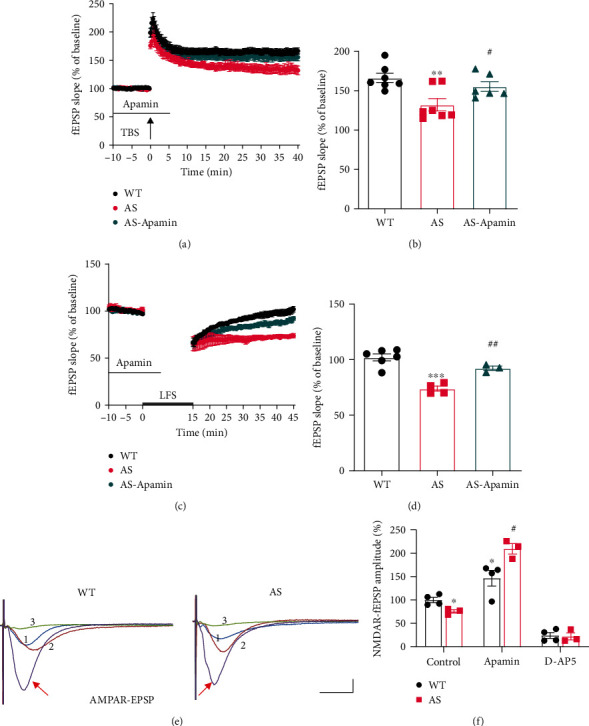
Impairment of NMDA receptor-dependent hippocampal synaptic transmission and activity-dependent synaptic plasticity in hippocampal slices from female AS mice. (a) Reversal of LTP impairment in AS mice by SK2 channel blocker, apamin. Slopes of fEPSPs were normalized to the average values recorded during the 10-min baseline. (b) Means ± SEM of fEPSPs measured 40 min after TBS in different groups. *N* = 6 − 7 slices from 4-5 mice, ^∗∗^*P* < 0.01, #*P* < 0.05 (one-way ANOVA followed by Dunnett's test, *F* (2, 17) = 7.122). (c) LTD in hippocampal slices from AS mice is blocked by apamin. (d) Means ± SEM of fEPSPs measured 45 min after LFS in different groups. *N* = 3 − 6 slices from 3 mice, ^∗∗∗^*P* < 0.001, ##*P* < 0.01 (one-way ANOVA followed by Dunnett's test, *F* (2, 10) = 25.50). (e) and (f) Effects of apamin treatment on NMDAR-mediated synaptic responses (NfEPSPs). (e) Representative traces. (1) Mg^2+^-free aCSF containing 10 *μ*M CNQX (control), (2) Apamin treatment, (3) D-AP5 treatment. Scale bar, 0.5 mV/10 ms. (f) Quantification of NfEPSPs. Data are means ± SEM expressed as % of values in WT hippocampal slices before apamin treatment (WT control). *N* = 3 − 4 slices from 3 mice, ^∗^*P* < 0.05, # *P* < 0.05 (two-way ANOVA followed by the Tukey test; for genotype, *F* (1, 15) = 2.126, for treatment, *F* (2, 15) = 115.8, for interaction, *F* (2, 15) = 9.899). Traces of AMPAR fEPSPs indicate that synaptic responses before initiation of NfEPSP recording are similar between AS and WT mice.

Previous experiments showed that theta-burst stimulation (TBS) in field CA1 of hippocampal slices from male AS mice elicited only a transient facilitation of synaptic transmission, which decayed rapidly, and LTP failed to consolidate [29, 37]. In female AS mice, the amplitude of TBS-induced LTP was also significantly reduced as compared to female WT mice (Figures 3(a) and 3(b)). We then determined whether SK2 activity contributed to reduce LTP by using a selective SK2 channel blocker, apamin. Following preincubation of hippocampal slices from female AS mice with apamin (20 nM), the magnitude of TBS-elicited LTP was significantly enhanced and almost reached the level seen in female WT mice (Figures 3(a) and 3(b)). We also analyzed long-term depression (LTD), another type of synaptic plasticity postulated to be involved in learning and memory, in hippocampal slices from female mice. Low-frequency stimulation (LFS; 1 Hz, 15 min) of the Schaffer collaterals in hippocampal slices from 2~3-month-old female WT mice induced a transient synaptic depression, and synaptic responses slowly returned to baseline levels (Figure 3(c)), a result consistent with the literature [37, 44]. However, the same protocol induced sustained LTD in slices from 2~3-month-old female AS mice, which is similar to what we previously reported in male AS mice [37]. Apamin (20 nM) pretreatment also significantly reduced LTD in female AS mice (Figures 3(c) and 3(d)).

Both TBS-induced LTP and LFS-induced LTD at Schaffer collateral-CA1 synapses are known to be dependent on NMDARs, and previous studies have demonstrated that SK2-mediated hyperpolarization inhibits NMDAR-channel opening [37]. We previously showed that, in male AS mice, NMDAR-mediated synaptic responses (NfEPSPs) were significantly reduced as compared to male WT mice, and that apamin application normalized NfEPSPs in male AS mice. We thus performed similar experiments in female AS mice. NfEPSPs were isolated by bath application of Mg^2+^-free aCSF containing 6-cyano-7-nitroquinoxaline-2, 3-dione (CNQX; 10 *μ*M) to block AMPA-receptor-mediated synaptic transmission. The NMDAR antagonist AP5 was used to verify that NfEPSPs were mediated by NMDARs under these conditions (Figures 3(e) and 3(f)). As previously observed in male AS mice, NfEPSP amplitudes were significantly lower in female AS mice as compared to female WT mice, and this decrease was reversed by apamin application (Figures 3(e) and 3(f)), suggesting that SK2 imposes a stronger inhibition of NMDAR in female AS mice than in female WT mice. Interestingly, apamin also significantly increased NfEPSP amplitude in female WT mice (Figures 3(e) and 3(f)), which is different from what we observed in male WT mice, where apamin did not affect NfEPSPs [37].

### 2.3. Apamin Treatment Improves Learning and Memory in Female AS Mice

**Figure 4:**
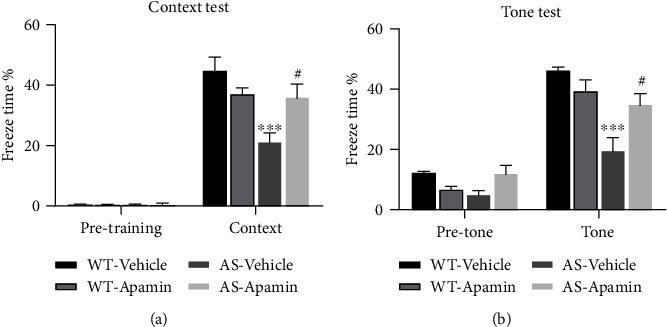
Effects of apamin treatment on fear conditioning in female WT and AS mice. WT and AS mice were treated with apamin (0.4 mg/kg) 30 min before training for fear-conditioning, as described in the Methods. Results are expressed as percent (%) time mice exhibited freezing behavior. (a) % freezing time for different experimental groups in context memory (means ± SEM of 6-8 mice; ^∗∗∗^*P* < 0.001, as compared to WT-Vehicle mice; #*P* < 0.05, as compared to AS-Vehicle mice (two-way ANOVA followed by the Tukey test; for genotype, *F* (1, 23) = 10.29, for treatment, *F* (1, 23) = 0.8259, for interaction, *F* (1, 23) = 8.708). (b) % freezing time for different experimental groups in tone memory (means ± SEM of 6-8 mice; ^∗∗∗^*P* < 0.001, as compared to WT-Vehicle mice; #*P* < 0.05, as compared to AS-Vehicle mice (two-way ANOVA followed by the Tukey test; for genotype, *F* (1, 23) = 16.17, for treatment, *F* (1, 23) = 1.229, for interaction, *F* (1, 23) = 8.343).

To determine whether apamin could also reverse impairment in hippocampus-dependent learning in female AS mice, we used the fear-conditioning paradigm. AS and WT female mice were injected with apamin (0.4 mg/kg, i.p.) 30 min before the training session, as previously reported [37, 41]. While there was no difference in freezing time in the preconditioning period, during training or before tone application in the testing period among all experimental groups (Figures 4(a) and 4(b)), female AS mice exhibited much less freezing time in both context-dependent (Figure 4(a)) and tone-dependent (Figure 4(b)) memory recall, as compared to WT female mice. Apamin treatment significantly enhanced memory recall in female AS mice (Figure 4). Under our experimental conditions (three conditioned stimuli (CS) paired with three unconditioned stimuli (US)), apamin treatment did not affect learning in female WT mice (Figure 4).

## 3. Discussion

Abnormal synaptic plasticity, with reduced LTP and enhanced LTD, has been repeatedly reported in hippocampal slices from AS mice [25, 27–29]. We previously showed that UBE3A-mediated ubiquitination and subsequent degradation of synaptic SK2 channels play critical roles in synaptic plasticity and learning and memory. In male AS mice, the lack of UBE3A resulted in increased synaptic SK2 levels, which contributes to abnormal synaptic plasticity and impairment in learning and memory in the fear-conditioning paradigm [37]. These findings are consistent with the literature [39–41]. In the present study, we showed that female mice also exhibited reduced TBS-induced LTP and enhanced LFS-induced LTD, as well as impairment in both contextual and auditory fear memory. Abnormal synaptic plasticity and memory impairment were markedly improved by treatment with apamin, a SK2 channel blocker, suggesting that increased SK2 channel activity is involved in the pathophysiology of AS mice. Indeed, like in male AS mice, electrophysiological studies revealed that increased SK2 levels imposed a tonic inhibition of NMDARs, accounting for the abnormal synaptic plasticity observed in AS mice.

Interestingly, there were also subtle differences between the present results and our previous results with male WT and AS mice. First, the reduction in LTP amplitude observed in female AS mice was not as severe as in male AS mice, as synaptic responses did not return to baseline at 40 min post TBS. Second, apamin, used at the same concentration in both studies, did not affect NMDAR fEPSP amplitude in hippocampal slices from male WT mice [37, 38], but significantly increased it in female WT mice. It has been previously reported that LTP in hippocampal CA1 region is more difficult to elicit in female than in male rodents [43, 45]. It is tempting to speculate that SK2 channels impose a stronger inhibition of NMDAR in female than in male WT mice, which could increase the threshold for LTP induction in CA1 region of female mice. To overcome the higher threshold for and facilitate LTP induction in females, an additional mechanism is therefore required. In a study with rodents, Wang et al. [43] showed that activation of ER*α* through locally produced estrogen is necessary for LTP induction in females but not in males. The molecular signaling downstream of estrogen receptors is not completely clear and may involve metabotropic NMDAR activity and activation of ERK, PKA, mTOR, and other signaling pathways [46, 47]. Of note, we previously showed that although one TBS failed to induce LTP, two TBS applied within 45 min were able to induce LTP in AS mice [37]. We subsequently showed that the effect of two TBS on LTP was due to PKA activation-induced SK2 endocytosis, as it was blocked by a PKA inhibitor and occluded with apamin [38]. Our recent study also showed that although TBS-induced LTP was impaired in male AS mice, high-frequency stimulation-(HFS-) induced LTP was not [48]. We also previously showed that, while TBS-induced LTP was ERK-dependent, HFS-induced LTP relied more on PKA activation [49]. These results imply that PKA activation facilitates LTP in male AS mice. Whether the same mechanism also holds true in female mice remains to be determined, but if it were, estrogen-mediated PKA activation could account for the differences in LTP between male and female AS mice. Along this line, to the best of our knowledge, sex differences in the expression and regulation of SK2 channels in the CNS remain unsettled, although sexual dimorphism has been reported in cardiac SK channel activation and regulation [50]. Additionally, SK2 channels have been shown to influence neuronal intrinsic plasticity through regulation of membrane excitability [51, 52]. Whether these additional roles of SK2 channels also contribute to alterations in synaptic plasticity and memory in AS mice, this remains to be determined.

Despite these subtle differences in synaptic plasticity, female AS mice exhibited similar impairment in fear-conditioning learning as male AS mice. A recent study specifically designed to address sex differences in AS mice [36] showed that, during the training/conditioning phase, male AS mice exhibited enhanced responses, and female AS mice showed reduced responses to shocks when compared to their respective WT mice. Both male and female AS mice exhibited impairment in fear memory recall 24 h after conditioning, however, there was no sex difference. This study further showed that the sex differences observed in training responses were due to altered pain sensation, with male AS mice exhibiting enhanced pain sensation while female AS mice had reduced pain sensation, when compared to their respective WT mice. There was no difference between WT and AS when male and female mice were combined. This finding is different from a previous report showing that AS mice had enhanced nociception as compared to WT mice without sex differences. We did not observe any sex or genotype differences in freezing responses during training, which is consistent with previously published data [28, 32]. Additionally, experimental settings, animal age, and genetic background were different between our studies and Koyavski et al.'s report [36]. We used three tone-footshock pairings with a 2 s shock at 0.75 mA in 3-4 month-old 129 mice, while Koyavski et al.'s study used two 2 s foot-shocks at 0.5 mA in 5-8 month-old C57BL/6 J mice. Both age and strain backgrounds have been shown to influence the degree of behavioral deficiency of AS mice [26, 34]. Nevertheless, the general conclusion seems to be that there is no sex difference in fear-conditioning learning and that both male and female AS mice are equally impaired. Another recent study using combined males and females reported that, while AS mice performed poorly in spatial learning in the Morris water maze in massed training, learning was improved in a spaced training paradigm [53]; no sex difference was reported. It is therefore possible that downregulation of SK2 by PKA-mediated phosphorylation of SK2 induced by a second TBS or by HFS [48] contributes to the improved performance observed in spaced learning. Koyavski et al.'s [36] study also compared female and male WT and AS mice in the Morris water maze spatial learning paradigm. While they confirmed that AS mice performed much worse than WT mice, there were no significant sex differences within the genotype.

There are a couple of shortcomings in the current study. First, the study used only female WT and AS mice and did not include a direct comparison of male and female mice. Second, we did not identify female mice in the different phases of the estrous cycle (proestrous, estrous, and metestrous). Nevertheless, our results support the general conclusion previously reported in the field that there is no clear sex difference in fear-conditioning learning in AS mice. These results support the observation that there are no marked sex differences regarding major cognitive functions in human AS subjects, while there might be subtle sex differences in other phenotypic traits. Along this line, it has been recently reported that a 6-week environmental enrichment period restored motor coordination, marble burying, and forced swim behavior in male AS mice to the level of WT mice, while having no effect on female AS mice [54]. Results from our study also provided a molecular mechanism for the observed benefit of spaced training as a potential therapeutic strategy for enhancing cognition in AS.

## 4. Methods

### 4.1. Animals

Animal experiments were conducted in accordance with the principles and procedures of the National Institutes of Health Guide for the Care and Use of Laboratory Animals. All protocols were approved by the Institutional Animal Care and Use Committee of Western University of Health Sciences. Original UBE3a mutant (AS) mice were obtained from The Jackson Laboratory, strain B6.129S7-UBE3atm1Alb/J (stock No. 016590), and a breeding colony was established, as previously described 30. In all experiments female AS mice aged between 2–4 months were used. Control mice were age-matched, female, and wild-type littermates. Multiple liters were used. Mice, housed in groups of two to three per cage, were maintained on a 12-h light/dark cycle with food and water ad libitum.

### 4.2. P2/S2 Fractionation and Western Blots

P2/S2 fractionation and western blots were performed according to published protocols [27]. Briefly, hippocampal tissue or slices were homogenized in ice-cold HEPES-buffered sucrose solution (0.32 M sucrose, 4 mM HEPES, pH 7.4) with protease inhibitors. Homogenates were centrifuged at 900 g for 10 min to remove large debris (P1). The supernatant (S1) was then centrifuged at 11,000 g for 20 min to obtain crude membrane (P2) and cytosolic (S2) fractions. P2 pellets were sonicated in RIPA buffer (10 mM Tris, pH 8, 140 mM NaCl, 1 mM EDTA, 0.5 mM EGTA, 1% NP-40, 0.5% sodium deoxycholate, and 0.1% SDS). Protein concentrations were determined with a BCA protein assay kit (Pierce). The samples were separated by SDS-PAGE and transferred onto a PVDF membrane (Millipore). After blocking with 3% BSA for 1 h, membranes were incubated with specific antibodies (anti-SK2 antibody, Alomone Labs, 1 : 500; anti-b-actin, Sigma-Aldrich, A5441, 1 : 1000; anti-UBE3A, Bethyl, A300-351A, 1 : 2000) overnight at 4°C followed by incubation with secondary antibodies (IRDye secondary antibodies; Li-COR) for 2 h at room temperature. Antibody binding was detected with the Odyssey® family of imaging systems.

### 4.3. Immunofluorescence of Brain Tissue Sections

Deeply anesthetized animals were perfused, and brains were postfixed in 4% PFA overnight followed by sequential immersion in 15% and 30% sucrose for cryoprotection. Brains were then sectioned (20 *μ*m) and stained as previously described [27]. Briefly, sections were blocked in 0.1 M PBS containing 5% goat serum and 0.3% Triton X-100, and then incubated in primary antibody mixture including rabbit anti-SK2 (1 : 200; APC-028, Alomone Labs) and mouse anti-PSD95 (1 : 200; MA1-045, Invitrogen) in 0.1 M PBS containing 1% BSA and 0.3% Triton X-100 overnight at 4°C. Sections were washed 3 times (10 min each) in PBS and incubated in appropriate Alexa Fluor–conjugated secondary antibodies (Invitrogen) for 2 h at room temperature. Images were acquired using a Zeiss LSM880 AiryScan confocal microscope.

### 4.4. Image Analysis and Quantification

Images for all groups were obtained using identical acquisition parameters and analyzed using Zen (Zeiss) and ImageJ (NIH) software. In all cases the experimenter was blind regarding the identity of the samples during acquisition and analysis. All immunostaining studies were performed in at least three independent experiments. The apical dendrites in the CA1 region of hippocampus were randomly selected for puncta analysis. Four-six mice were used for each genotype; 6-8 regions of interest and over 500 particles were analyzed for each mouse. The intensity and number of SK2- and PSD95-stained puncta were quantified. Colocalization analysis by Mander's coefficients was performed using Zen.

### 4.5. Acute Hippocampal Slice Preparation and Electrophysiology

Acute hippocampal transversal slices (400 *μ*m-thick) were prepared from adult female mice (2-4-month-old) as previously described [29], and recording was done in an interface recording chamber; slices were continuously perfused with oxygenated (95% O_2_/5% CO_2_) and preheated (33 ± 0.5°C) artificial cerebrospinal fluid (aCSF) (in mM) [110 NaCl, 5 KCl, 2.5 CaCl_2_, 1.5 MgSO_4_, 1.24 KH_2_PO_4_, 10 D-glucose, 27.4 NaHCO_3_]. Field EPSPs (fEPSPs) were elicited by stimulation of the Schaffer collateral pathway in CA1 stratum radiatum. Before each experiment, the input/output (I/O) relation was examined by varying the intensity of the stimulation. Long-term potentiation was induced using theta-burst stimulation (10 bursts at 5 Hz, each burst consisting of 4 pulses at 100 Hz). Long-term depression was induced by 900 pulses delivered at 1 Hz. Synaptic NMDA receptor-mediated responses were obtained using Mg^2+^-free aCSF containing 10 *μ*M CNQX. Data were collected and digitized by Clampex; the slope of fEPSP was analyzed in most of the experiments, except for NMDA receptor-mediated responses in which the amplitude of fEPSP was analyzed.

### 4.6. Fear Conditioning

AS mice and their WT littermates were randomly assigned to either drug or vehicle groups and blinded to the examiner. Mice were injected intraperitoneally (i.p.) with apamin (0.4 mg/kg body weight) 30 min before being placed in the fear-conditioning chamber (H10-11-M-TC, Coulbourn Instruments). The conditioning chamber was cleaned with 10% ethanol to provide a background odor. A ventilation fan provided a background noise at ∼55 dB. After a 2-min exploration period, three tone-footshock pairings separated by 1-min intervals were delivered. The 85 dB 2 kHz tone lasted 30 s and coterminated with a footshock of 0.75 mA and 2 s. Mice remained in the training chamber for another 30 s before being returned to home cages. Context test was performed one day after training in the original conditioning chamber with 5 min recording. On day three, animals were subjected to cue/tone test in a modified chamber with different texture and color, odor, background noise, and lighting. After 5 min recording, mice was exposed to a tone (85 dB, 2 kHz) for 1 min. Mouse behavior was recorded with the FreezeFrame software and data were analyzed using the FreezeView software (Coulbourn Instruments). Motionless bouts lasting more than 1 s were considered as freezing. The percent of time animal froze was calculated, and the group means with SEM and cumulative distribution of % freezing were analyzed.

### 4.7. Statistical Analysis

All data are expressed as means ± SEM. To compute *P* values, unpaired Student's t-test, one-way and two-way ANOVA with Dunnett's or Tukey's post-test were used (GraphPad Prism 8), as indicated in the figure legends. The level of statistical significance was set at *P* < 0.05.

## Data Availability

The data supporting the results of the study can be obtained by request.
